# The Impact of the Addition of a Physical Therapy Assistant to the Treatment Team for Management of Neck Pain: A Retrospective Analysis of Outpatient Physical Therapy Clinics

**DOI:** 10.7759/cureus.42751

**Published:** 2023-07-31

**Authors:** Anthony N Baumann, Deven P Curtis, Mingda Chen, Keith D Baldwin

**Affiliations:** 1 Department of Rehabilitation Services, University Hospitals, Cleveland, USA; 2 College of Medicine, Northeast Ohio Medical University, Rootstown, USA; 3 College of Medicine, Case Western Reserve University, Cleveland, USA; 4 Division of Orthopaedics, Children's Hospital of Philadelphia, Philadelphia, USA

**Keywords:** cervicalgia, neck pain, rehabilitation, physical therapy, physical therapy assistant

## Abstract

Introduction

The impact of physical therapy assistants (PTAs) on patient outcomes, mostly in the acute and subacute setting, is well known in the literature. However, no study to date has examined the impact of using PTAs as part of a treatment team in the outpatient setting for common musculoskeletal conditions. The purpose of this study is to determine if physical therapy team composition, either physical therapists (PTs) only or a team consisting of PTs and PTAs, has a significant impact on patient outcomes in adult patients with musculoskeletal neck pain to help investigate an ideal practice pattern for outpatient physical therapy.

Methods

This is a retrospective cohort study analyzing the impact of physical therapy treatment team composition (PTs only, or team consisting of PTs and PTAs) on pain, active range-of-motion (AROM), and disability outcomes via the Neck Disability Index (NDI) in the conservative treatment of neck pain. All patients were treated with usual physical therapy care. Inclusion criteria involved patients with a diagnosis of neck pain (M48.2), older than 18 years old, a physical therapy evaluation procedure code (97161, 97162, 97163), and at least two visits per bout of physical therapy. Primary outcome measures were pain, bilateral rotation AROM, disability, and number of visits.

Results

Included patients (n=195) had an average age of 60.8 years ± 16.1 years with an average number of total physical therapy visits of 7.4 visits ± 4.3 visits (range, 2 visits - 22 visits) with 120 patients (61.5%) treated by a PT only (PT-only group) and 75 patients (38.5%) treated by a team consisting of a PT and a PTA (PTA group). The PT-only group had significantly fewer visits than the PTA group (p<0.001). The PT-only group had a pain improvement of 2.1 points ± 2.3 points whereas the PTA group had a pain improvement of 2.2 points ± 2.4 points with no significant difference between the two groups (p=0.573). The PT-only group (n=46 patients) had an average rotation AROM improvement of 20.0 ± 17.4 degrees whereas the PTA group (n=40 patients) had an average rotation AROM improvement of 16.8 degrees ± 23.0 degrees with no significant difference between the level of rotation AROM improvement between the two groups (p=0.408). Furthermore, there was also no significant difference in the amount of NDI improvement seen in both groups (p=0.594).

Conclusion

There was no significant difference in patient outcomes for pain, AROM, and disability when PTAs were added to the physical therapy treatment team in the conservative management of neck pain in the outpatient setting. However, patients treated with a treatment team consisting of PTAs had significantly more visits, despite no significant change in outcomes. Randomized controlled trials are needed as the reasons for these findings can be many and require further research.

## Introduction

Treatment team composition is a topic of increasing interest in the medical field, as numerous studies have examined the impact of advanced practice providers (APPs) working with physicians on overall patient outcomes and healthcare cost [1-6]. Similar questions have been asked in the field of physical therapy as to the value and impact of physical therapy assistants (PTAs) on patient outcomes, mostly in the setting of skilled nursing facilities (SNFs) and acute care [7-10]. Recent changes in Medicare policies have impacted physical therapy staffing in SNFs, with greater impact on the staffing of PTAs [9]. Although a great deal of research has focused on the utility of PTAs in those settings on pathologies such as cerebrovascular accidents (CVAs), no study to date has examined the impact of PTAs on patient outcomes for common musculoskeletal pathologies, such as neck pain, in the outpatient setting [7].

Neck pain is a common musculoskeletal pathology frequently treated by physical therapists (PTs) and PTAs in the outpatient clinical setting [11-13]. The need to improve quality of care while decreasing costs is increasingly relevant in the conservative treatment of neck pain as up to 70% of individuals will have neck pain at some point [11-13]. While studies examining the utilization of PTAs in other settings have reported improved outcomes, such as increased community discharges, the nature of the outpatient physical therapy treatment of neck pain poses new variables that impact generalizability of findings in the acute and sub-acute setting to the outpatient setting [7,9]. For example, manual therapy is a recommended component of physical therapy for neck pain, for which PTAs may have limited exposure due to education as compared to PTs [12,13]. Therefore, it remains to be seen if the utilization of PTAs in the outpatient setting significantly impacts patient outcomes when treating neck pain.

The hypothesis of this study is that a treatment team composed of only a PT, which has a higher education level than a PTA and possibly more exposure to treatments recommended by clinical practice guidelines (CPGs), will have superior outcomes for pain, active range-of-motion (AROM), and disability when treating patients with neck pain [12,13]. The purpose of this study is to assess the impact of physical therapy team composition (PTs only vs PTs and PTAs) on patient outcomes for adult patients presenting with neck pain in an outpatient setting.

## Materials and methods

Study creation

This is a retrospective cohort study analyzing the impact of physical therapy treatment team composition (PTs only or team consisting of PTs and PTAs) on pain, AROM, and disability outcomes in the conservative treatment of neck pain. All patients were treated with usual physical therapy care and specific interventions given to each patient were not controlled or measured for this study. This study was performed in accordance with the first author’s Institutional Review Board at University Hospitals (STUDY # 20220552).

Inclusion and exclusion criteria

Patient records were obtained from multiple outpatient clinics within a single hospital system from 2018 to 2022. Records were obtained with the help of the first author’s institutional research center. Inclusion criteria were patients with a diagnosis of neck pain (M48.2), older than 18 years old, and a physical therapy evaluation procedure code (97161, 97162, 97163). Patients were excluded if they did not have a diagnosis of neck pain, had a history of neck surgery, or had less than two physical therapy visits in total throughout their bout of physical therapy. Patients had to have complete pre- and post-physical therapy pain scores for study inclusion, but patients were included even if they didn’t have complete information for AROM and disability due to the potential of performing subgroup analysis.

Study definitions

Patients were stratified into the PT-only group or the PTA group dependent on the presence or absence of treatment by a PTA during the bout of physical therapy. For the purpose of this study, the “PT-only group” refers to patients that were only treated by a PT and were not treated by a PTA at any point during their bout of physical therapy. On the contrary, the “PTA group” refers to patients that were treated by a team of a PT as well as a PTA. To qualify for inclusion into the PTA group, a patient had to be treated by a PTA at least once during the bout of physical therapy. ROM was measured via goniometer in this study as a summative measure of both right and left cervical rotation in degrees. Neck Disability Index (NDI) percentage score is expressed as a percentage out of 100, with 0% representing no disability and 100% representing total disability. The number of physical therapy visits per bout of physical therapy was used as a proxy for follow-up time. Usual physical therapy care is defined as any outpatient physical therapy treatment frequently given to patients at the discretion of the treating clinician and includes common interventions such as manual therapy, exercise, and modalities (ice, heat, and electrical stimulation). Usual physical therapy care was used in this study to mimic "real-world" physical therapy care that is highly variable and dependent on the PT. 

Data extraction

Data extraction was completed by multiple authors during the course of this study. Data collected during this study were patient age, sex, duration of symptoms (days), pre- and post-physical therapy pain (measured on a 0-10 Visual Analog Score), pre- and post-physical therapy disability (measured via the Neck Disability Index percentage), pre- and post-physical therapy cervical rotation AROM, total number of physical therapy visits, and total number of visits performed by a PTA in the PTA group.

Statistical analysis

This study used SPSS version 29.0 (IBM Corp., Armonk, NY, USA) for statistical analysis. Frequency count and descriptive statistics were used to describe the demographics of the patient cohort. The Kolmogorov-Smirnov or the Shapiro-Wilk test was used to test the distribution of the data based on sample size. For comparisons between two groups, an independent samples t-test or Mann Whitney U test was used depending on the nature of the data. For this study, p-values were considered significant at 0.05.

## Results

Entire cohort demographics

The entire study cohort (n=195 patients) had an average age of 60.8 years ± 16.1 years with an average number of total physical therapy visits of 7.4 visits ± 4.3 visits (median: 7.0 visits; range, 2 visits - 22 visits). Out of the total cohort, 167 patients (85.6% of patients) reported duration of symptoms with a mean duration of symptoms in days of 245.4 days ± 374.7 days (median: 110.0 days; range, 3 days - 2,500 days). Of the 167 patients with reported duration of symptoms, 105 patients (62.9% of patients) had neck pain for less than 180 days and 62 patients (37.1%) had neck pain for more than 180 days. Refer to Table 1 for more information on cohort demographics.

**Table 1:** Demographic information for the entire cohort of patients included in this study. Abbreviations: SD, standard deviation.

Entire cohort demographic information	Values
Patients (n,%)	195 (100%)
Mean ± SD age, years	60.8 ± 16.1
Median age, years	64.0
Mean ± SD total visits, number of visits	7.4 ± 4.3
Median total visits, number of visits	7.0
Mean ± SD duration of symptoms, days	245.4 ± 374.7
Median duration of symptoms, days	110.0
Acute/subacute neck pain (n, %)	105 (62.9%)
Chronic neck pain (n, %)	62 (37.1%)

Group demographics

From the entire cohort (n=195 patients), 120 patients (61.5%) were treated by a PT only (PT-only group) whereas 75 patients (38.5%) were treated by a team consisting of a PT and a PTA (PTA group). The PT-only group (n=120 patients) had a mean age of 58.9 years ± 16.1 years and the PTA group (n=75 patients) had a mean age of 64.0 years ± 15.6 years, with the PT-only group containing patients with a significantly lower age (p=0.024). For total number of visits for each group, the PT-only group (n=120 patients) had a mean visit number of 6.8 visits ± 4.5 visits whereas the PTA group (n=75 patients) had 8.3 visits ± 3.8 visits, with the patients in the PT group having significantly fewer visits (p<0.001). The PTA group had an average of 3.6 PTA visits ± 2.3 PTA visits per patient. Refer to Table 2 for information on group demographics.

**Table 2:** Demographic information for both groups included in this study. Abbreviations: SD, standard deviation.

Group demographic information	Physical therapy only group	Physical therapy assistant group	Between group p-value
Patients (n, %)	120 (61.5%)	75 (38.5%)	-
Mean ± SD age, years	58.9 ± 16.1	64.0 ± 15.6	p=0.024
Median age, years	63.0	65.0	
Mean ± SD total visits, years	6.8 ± 4.5	8.3 ± 3.8	p<0.001
Median total visits, years	5.0	8.0	
Mean ± SD physical therapy assistant visits, number of visits	-	3.6 ± 2.3	-
Median physical therapy assistant visits, number of visits	-	3.0	-

Pain outcomes

There was no significant difference in the pre-pain levels between the PT-only group and the PTA group (p=0.961). The PT-only group had a mean pre-pain level of 4.0 ± 2.4 points and a mean post-pain level of 1.9 ± 2.0 points. The PTA group had a mean pre-pain level of 4.0 points ± 2.8 points and a mean post-pain level of 1.8 ± 2.1 points. The PT-only group had a mean pain improvement of 2.1 points ± 2.3 points (median: 2.0 points) whereas the PTA group had a mean pain improvement of 2.2 points ± 2.4 points (median: 2.0 points) with no significant difference between the two groups (p=0.573). Both groups demonstrated a clinically significant improvement in pain (greater than 1.37/10 points) on the Visual Analog Score based on the minimally clinical important difference (MCID) used in other orthopedic studies [11,14]. However, as noted above, it should be noted that the PTA group had significantly more visits as compared to the PT-only group with no significant difference in pain improvement. Refer to Table 3 below for information on pain outcomes.

**Table 3:** Improvement in pain, active range-of-motion, and disability for each group included in this study. Abbreviations: SD, standard deviation; AROM, active range-of-motion; NDI, Neck Disability Index

Categories	Physical therapy only group	Physical therapy assistant group	Between group p-value
Mean ± SD pain improvement, points	2.1 ± 2.3	2.2 ± 2.4	p=0.573
Median pain improvement, points	2.0	2.0	
Subjects (n,%)	120 (100%)	75 (100%)	-
Mean ± SD rotation AROM, degrees	20.0 ± 17.4	16.8 ± 23.0	p=0.408
Median rotation AROM, degrees	20.0	16.5	
Subjects (n,%)	46 (38.3%)	40 (53.3%)	-
Mean ± SD NDI disability score, percentage score	8.5 ± 9.6	9.8 ± 10.2	p=0.594
Median NDI disability score, percentage score	8.0	10.0	
Subjects (n,%)	40 (33.3%)	32 (42.6%)	-

Active range-of-motion outcomes

For subgroup analysis for AROM outcomes, a total of 86 patients had complete pre-physical therapy and post-physical therapy AROM outcomes with 46 patients in the PT-only group and 40 patients in the PTA group. There was no significant difference in the pre-AROM levels between the PT-only group and the PTA group (p=0.358). The PT-only group had a mean pre-AROM of 106.4 degrees ± 21.7 degrees and a mean post-AROM of 126.5 degrees ± 22.0 degrees. The PTA group had a mean pre-AROM of 101.8 degrees ± 24.5 degrees and a mean post-AROM of 118.6 degrees ± 23.3 degrees. The PT-only group (n=46 patients) had an average AROM improvement of 20.0 ± 17.4 degrees whereas the PTA group (n=40 patients) had an average AROM improvement of 16.8 degrees ± 23.0 degrees. There was no significant difference between the level of AROM improvement between the two groups (p=0.408). However, both groups had a relatively large improvement in cervical AROM as the average pre-AROM was 106.4 degrees ± 21.7 degrees and 101.8 degrees ± 24.5 degrees for the PT-only group and PTA group, respectively. Refer to Table 3 for information on ROM outcomes for both groups.

Disability outcomes

For subgroup analysis for disability outcomes using the Neck Disability Index, a total of 72 patients had complete pre-physical therapy and post-physical therapy NDI scores (expressed as a percentage out of 100, with 0 representing no disability and 100 representing total disability). The PT-only group had 40 patients and the PTA group had 32 patients. There was no significant difference in pre-physical therapy NDI levels between the PT-only group and the PTA group (p=0.312). The PT-only group had a mean pre-physical therapy NDI percentage score of 24.6% ± 14.1% and a mean post-physical therapy NDI percentage score of 16.1% ± 13.7%. The PTA group had a mean pre-physical therapy NDI percentage score of 28.6% ± 16.5% and a mean post-physical therapy NDI percentage score of 18.8% ± 17.4%. There was also no significant difference in the amount of NDI improvement seen in both groups (p=0.594). Both groups had clinically insignificant improvements in NDI percentage score with the PT-only group improving by 8.5% ± 9.6% and the PTA group improving by 9.8% ± 10.2% as the MCID is 19% for the NDI [15]. Refer to Table 3 for information on disability outcomes for both groups.

## Discussion

The relevance of physical therapy team composition on patient outcomes when treating neck pain in the outpatient setting is evident due to high economic burden of neck pain as well as similar questions of personnel utilization in other fields of medicine [1-6,8,9,12,13]. While the literature has examined this question about the value and utilization of PTAs in the acute and subacute care settings, this study is the first article to date on the impact of PTA utilization on patient outcomes for musculoskeletal conditions in the outpatient physical therapy clinic [7-10]. The hypothesis of this study was not upheld as this study demonstrated that the addition of PTAs to a physical therapy team in the outpatient setting did not adversely impact patient outcomes for neck pain in terms of pain, ROM, and disability as compared to physical therapy teams composed of PTs alone.

Based on the results of this study, the PT-only group improved by 8.5% ± 9.6% on the NDI and the PTA group improved by 9.8% ± 10.2% on the NDI, indicating clinically insignificant results as the MCID for the NDI is 19% [15]. Therefore, while there was no adverse impact on disability outcomes via the addition of a PTA, neither group experienced clinically significant improvement in disability. There was also no significant difference in pain outcomes between groups with the PT-only group having a mean pain improvement of 2.1 points ± 2.3 points (median: 2.0 points) and the PTA group having a mean pain improvement of 2.2 points ± 2.4 points (median: 2.0 points)(p=0.573). Likewise, it is important to note that both groups had clinically significant improvements in pain (greater than 1.37/10 points) per the MCID of the Visual Analog Scale used for other orthopedic conditions [11,14]. The authors are not aware of any MCID values for cervical rotation AROM; therefore, the ability to determine the clinical significance of this improvement is limited in this study. Interestingly, although there was no significant difference in any of the patient outcomes, the PT-only group utilized significantly fewer visits than the PTA group.

These findings are relevant as they factor into personnel decisions made by both private practices and large healthcare system organizations, as recent changes in Medicare continue to impact the field of physical therapy [8,10]. Unfortunately, the results of this study are unable to comment on the cost-to-benefit ratios of a team composed of PTs and PTAs as compared to PTs alone, but the question is raised as to the ideal physical therapy team composition. As the educational standard for physical therapists has progressed to a doctoral degree in recent years, the level of education between PTs and PTAs has grown as a consequence [16,17]. There is limited data on how this increased level of education impacts patient outcomes [17]. Caution should be taken with the results of this study as equal outcomes between the two therapy team options does not mean that PTs provide the exact same care as PTAs.

Indeed, this question requires further research as the results of this study could indicate that PTs are properly utilizing the skills of PTAs in their treatment plan. While some PTAs may be unfamiliar with interventions currently recommended by the CPGs for neck pain, such as dry needling and thoracic manipulation, the PTs in the PTA team may be utilizing the PTAs only when certain interventions within the skillset of the PTAs, such as exercise and resistance training, are more appropriate [12,13,18]. As all patients in this study received usual physical therapy care, which is highly variable, may not be consistent with the CPGs, and is at the discretion of the evaluating PTs, the specific interventions used by both of the treatment teams were not controlled for in this study [13,18,19]. Furthermore, it is also possible that even if the PTA team is less adherent to the interventions recommended in the CPGs due to the lack of specific skillsets, this may not have any significant impact on patient outcomes [12,19,20]. More research is needed to determine how the utilization of specific interventions via different physical therapy team compositions impacts patient outcomes, as this study was not able to control for this potentially confounding factor. One possibility for a future study may be to examine the impact of physical therapy treatment team composition for common musculoskeletal conditions for which the evidence-based treatments are more homogenous, such as shoulder pain.

There are multiple limitations of this study that impact generalization and application of the results. First, this study is retrospective in nature and contains bias in the form of patient selection. Patients were only included in the study if they continued with physical therapy for more than one visit and if they had complete pre- and post-physical therapy pain scores, thus implying selection bias. Future studies should focus higher level of evidence studies of a prospective nature to determine if the addition of a PTA to a physical therapy team has any impact on patient outcomes. Neck pain itself has varying etiologies, possibly confounding results. Also, the sample size of this study was relatively small, especially for subgroup analyses for ROM and disability. However, none of the results were close to approaching significance, but it is possible that significance could have been seen with a larger sample size. Therefore, it is important that future studies incorporate a larger sample size.

This study is also limited in terms of generalizability as all patients were from multiple clinics within a single hospital system within a single region of the United States. Therefore, the results of this study may not be generalizable to findings throughout the United States and the globe. Unfortunately, the PTA and the PT-only groups were not equal in terms of patient age and number of physical therapy visits, which could also impact outcomes. However, the authors believed that our hypothesis that the PT-only group would have greater improvements in outcomes would have been more easily seen in the younger cohort of the PT-only group, which was not the case. Furthermore, the significance in the number of visits for each group is a relevant finding as it is possible that the PTA group required more visits in order to achieve the same results as the PT-only group. This speculation is not proven by our study and needs further elucidation by future studies. This study is also unable to comment on how treatment team composition, visit utilization, and outcomes impact healthcare cost in outpatient clinics. Another limitation is the fact that the symptom duration of neck pain was not controlled for in this study and future research should investigate how symptom duration impacts outcomes in terms of treatment team composition. Finally, this study is meant to spur future research into the concept of ideal physical therapy treatment team composition to minimize healthcare cost, maximize patient outcomes, and improve overall patient satisfaction with physical therapy treatment for neck pain.

## Conclusions

The presence or absence of a PTA in a physical therapy treatment team did not significantly impact patient outcomes in terms of pain, cervical rotation AROM, or overall disability in the conservative treatment of neck pain. The findings of this study cautiously support the idea that the addition of a PTA to a physical therapy treatment team does not adversely impact patient outcomes, although the exact mechanism remains unknown and many confounding factors about specific patient treatments could impact these results. More research is needed on this topic to determine cost of the treatment team, timing, and overall impact on patient outcomes to obtain an ideal model for outpatient physical therapy treatment of neck pain. Finally, more research is also needed to see if these findings are present in other diagnoses, such as shoulder pain or hip pain, which are commonly treated in the outpatient physical therapy setting. 
